# When is deceased organ donation suitable? A critical reflection on medical criteria

**DOI:** 10.12669/pjms.35.2.570

**Published:** 2019

**Authors:** HR Ahmad, SG Haider, Adib Rizvi, Anwar Naqvi

**Affiliations:** 1 *HR Ahmad, Sindh Institute of Urology and Transplantation Karachi, Pakistan*; 2 *SG Haider, Sindh Institute of Urology and Transplantation Karachi, Pakistan. Faculty of Medicine, Heinrich-Heine University, Düsseldorf, Germany*; 3 *Adib Rizvi, Sindh Institute of Urology and Transplantation Karachi, Pakistan*; 4 *Anwar Naqvi, Sindh Institute of Urology and Transplantation Karachi, Pakistan*

**Keywords:** Energy, Electrical Impulses, Brain Death, Deceased Organ Donation

The overall trend of living organ donation, LOD is gradually shifting towards to deceased organ donation, DOD [Hoste et al 2016, Rodrigue et al 2016].^1^,^2^ DOD in China is currently reported to be 95% of the cases of transplantation treatment of organ failure. This contrasts with India and Pakistan where the main treatment of choice is through LOD [Chapman Jeremy, personal communication]. The percentage survival of renal graft at 1, 5 and 10 year in Australia has been reported to be 95, 85 and 75% respectively. USA figures are reported to be 90, 80 and 50% [O’Connell et al 2016].^3^ SIUT stands out remarkably well in the third world setting with one and 5-year graft survival of 92 and 85% of 3000 LOD transplants respectively [Rizvi et al. Am J Trans 2011].^4^ Why Pakistan is lagging behind in DOD? How the awareness could be awakened and improved for DOD?

Brain, heart, lungs and kidneys interalia are essential organs. Whenever there is a blood loss in a road accident, blood flow to brain, heart and lungs is maintained by the sympathetic drive but at the cost of temporary shutdown of blood supply to the rest of organs. As soon as this blood centralisation period is expired, the multiorgan failure leading to death can occur, if the point-of-care is not available to take over the initial body’s response to trauma [Ahmad and Hashmi 2018].^5^ The cerebral hypoxia switches off the sympathetic drive to set the body in a vicious cycle of failure. It looks like as if a machine has suddenly stopped working. Thus, the brain and heart function only when the energy supply is intact to generate and conduct electrical impulses via generation of action potentials by neurons. Likewise heart consumes energy to pump blood into vessels and lungs. All organs need the currency of energy in the form of an effectively recyclable intracellular molecule ATP. One may infer that these two energy and electrical impulses components finally hold the body alive. When energy cannot be supplied, death occurs. The human body without these two life lines is lifeless, cold and only a mass of material. It is cold because it lacks the energy of blood flow.

Before establishing the brain death of a patient, the possible confounders, e.g. coma and permanent vegetative state [PVS], should be completely excluded by the neurologists. Coma is a state that lacks both awareness and wakefulness [Ludwig et al 2016].^6^ The patient is unresponsive to pain stimuli. These patients should not be considered for the deceased organ donation, as the neurons are still generating action potentials, conducting electrical impulses and cells are producing ATP to keep the body alive. PVS is a stage when the patient may have awakened from COMA but still have not gained awareness. There is a complete lack of cognitive functions. The autonomic nervous system, located in the brainstem and in the thoracic spinal cord, is intact. Therefore, heartbeat, heart rhythms, pulmonary functions, renal and GIT functions are intact. The patients are kept with clinically assisted nutrition and hydration. They need a feeding tube but not a heart and lung machine. The patients may open their eyes in response to feeding. This state is also described as unresponsive wakeful syndrome. This state has been designated also as “Coma vigil” or PVS. These patients cannot be, and should not be included in the list of deceased organ donation as the generation of electrical impulses by neurons and the synthesis of ATP are still intact. Bottom line: The person is alive. The PVS patients can live in this state for many years. A famous case is that of Ariel Sharon. At the age of 76 in January 2006 he got a brain stroke. This led him into PVS. He was kept in the university hospital in this state up till January 2014 with a period of complete eight years. Then he died at the age of 84.

Brain death deals with the complete loss of the functions of cerebrum and brainstem. The definition of clinical brain death is well known. Using standard set of neurological tests and verifications, the brain death is determined by the absence of following functions: capacity for consciousness, centrally mediated motor responses, brain stem reflexes and capacity to breathe [Shemie and Baker, 2015].^7^ These clinical definitions have been included in the legal documents of Organ Transplantation Laws of many countries, in order to establish the brain death of a patient, suitable for organ donation. When two neurologists are being not involved in the treatment of the patients – after the standard tests and examination – come to the conclusion that the observations fulfil all clinical legal criteria, and therefore is here is a case of clinical brain death, then the patient or road accident victim should be considered for the deceased organ donation.

Therefore, the brain and circulatory death is ideal time to donate organs to enliven the suffering humanity on a long waiting list. This mode of organ transplantation can be made effective by looping units of critical care, neurology and surgery after raising the awareness, thinking and action of fellow citizens to come forward voluntarily to enliven humanity from suffering.
